# PCV2 Cap protein nuclear import via importin α/β receptor: molecular insights and antiviral potential

**DOI:** 10.3389/fmicb.2025.1701697

**Published:** 2025-11-19

**Authors:** Cui Lin, Na Li, Lihan Tao, Haiqin Li, Jiezhi Ma, Chengcheng Wu, Fanfan Zhang, Shaopei Fang, Jia Tan, Yezhen Huang, Xiaofei Zeng, Yanbing Zeng

**Affiliations:** 1 Institute of Animal Husbandry and Veterinary Medicine, Jiangxi Academy of Agricultural Sciences, Nanchang, Jiangxi, China; 2 Jiangxi Provincial Key Laboratory of Green and Healthy Breeding of Livestock and Poultry, Nanchang, Jiangxi, China; 3 Jiangxi Agriculture University, Nanchang, Jiangxi, China; 4 Jiangxi Provincial Animal Epidemic Prevention and Control Center, Nanchang, Jiangxi, China

**Keywords:** porcine circovirus type 2, capsid protein, nuclear transport receptors, importazole, ivermectin

## Abstract

Porcine circovirus type 2 (PCV2) Cap protein serves as the primary structural component and plays multiple roles in viral infection. Previous studies have shown that Cap predominantly localizes to the nucleus, but the molecular mechanisms governing its nuclear import remain unclear. This study demonstrates that PCV2 Cap undergoes an active nuclear transport process dependent on Ran-GTP and importin α, but not transportin-1. Co-immunoprecipitation assays revealed interactions between Cap and several host nuclear transport receptors (NTRs), including all importin α subunits, importin 4, 5, 7, and 11. Furthermore, the small molecule inhibitor importazole (IPZ) partially inhibits the nuclear translocation of Cap and suppresses Cap expression, as well as PCV2 replication. While ivermectin (IVM) only impedes transport without affecting viral replication. These findings provide significant insights into the molecular mechanisms of PCV2-Cap nuclear import and highlight potential therapeutic targets for antiviral strategies aimed at disrupting viral capsid transport.

## Introduction

Porcine circovirus type 2 (PCV2), the primary pathogen responsible for post-weaning multisystemic wasting syndrome (PMWS) in piglets, exhibits a broad prevalence range and rapid transmission rate, posing a significant threat to the swine industry (Hamel et al., 1998; Shulman and Davidson, 2017). The PCV2 virion, measuring approximately 17 nm in diameter, lacks an envelope and contains a single-stranded circular DNA genome. The complete PCV2 genome spans 1,766–1,768 nucleotides and is predicted to contain 11 open reading frames (ORFs) (Hamel et al., 1998). To date, only six ORF-encoded products have been confirmed to have known functions. Among these, the Rep and Rep’ proteins, encoded by *orf1*, serve as viral replicases (Cheung, 2012; Mankertz et al., 1998); the capsid protein Cap, encoded by *orf2*, functions as a structural protein and the major antigen inducing host immune responses (Trible et al., 2012), While other *orfs* encode non-essential proteins for viral replication, their functional roles in host interactions continue to be explored (He et al., 2013; Li D. et al., 2018; Lin et al., 2018; Liu et al., 2006; Lv et al., 2015).

The capsid protein (Cap) serves as the major structural component of PCV2 and plays a dual role in host immunity and viral pathogenesis, while also being essential for various stages of the viral life cycle. It has been reported that the N-terminal 41 amino acids of capsid comprise a nuclear localization signal (NLS), with two basic amino acid-rich regions at positions 12–18 and 34–41 playing critical roles in ensuring proper nuclear import (Liu et al., 2001). Furthermore, the fusion of this Cap NLS with the herpes simplex virus (HSV) peptide VP22 has been shown to enhance the cellular uptake of plasmid DNA, highlighting its potential as a carrier for facilitating DNA nuclear entry and targeted drug delivery (Chen et al., 2011). The spatiotemporal dynamics of Cap protein localization during PCV2 infection reveal that its nuclear accumulation is indispensable for viral replication, capsid formation, assembly and release processes (Cheung and Bolin, 2002; Finsterbusch et al., 2005). Regrettably, the molecular mechanisms governing PCV2 capsid and Cap protein translocation into the nucleus remain poorly understood.

The nuclear transport system serves as a critical regulatory mechanism mediating material exchange between the nucleus and cytoplasm. Proteins typically enter the nucleus through either passive diffusion or active transport, depending on their molecular weight. Active protein import into the nucleus is facilitated by multiple specialized mechanisms. Until recently, both classical and nonclassical nuclear import pathways have been discovered to assist in the nuclear transport of proteins. Only two classes of NLS have been biochemically and structurally characterized, the classical NLS (cNLS), which is recognized by the Importin-α/β heterodimer, and the PY-NLS (proline-tyrosine NLS), which is recognized by Transportin-1 (Soniat and Chook, 2015). The transport modes can be concisely described as the cNLS/importin α/importin β1 or the PY-NLS/transportin 1. The non-classical nuclear import pathway comprises diverse patterns of nuclear transport (Chook and Suel, 2011; Soniat and Chook, 2015). These processes involve three essential components: nuclear transport receptors (NTRs), which recognize and bind cargo proteins; RanGTP, which determines the direction of transport; and NLS, which serve as recognition motifs for transport factors (Jovanovic-Talisman and Zilman, 2017; Mackmull et al., 2017; Sorokin et al., 2007). In the coevolutionary relationship between viruses and hosts, viruses often exploit diverse nuclear import pathways to facilitate efficient infection. For instance, the influenza virus NP protein employs a classical import mechanism involving various importin α subunits (α1, α3, and α5) (Luo et al., 2018). HSV-1 VP19C exhibits non-classical NLS binding with importin β1 for nuclear import; as demonstrated by reduced replication efficiency in an NLS-mutated recombinant virus compared to wild-type HSV-1 (Li et al., 2012). Notably, the porcine circovirus Cap protein contains a nuclear localization sequence (NLS), yet the specific nuclear transport mechanism underlying its import remains unresolved.

In this study, we utilized fluorescence microscopy to demonstrate that the nuclear import of PCV2 Cap is mediated by a Ran-, importin α-, and importin β-dependent mechanism, excluding transportin-1. Furthermore, our findings revealed that both ivermectin (IVM) and importazole (IPZ) impaired the nuclear localization of Cap. While IPZ significantly reduced protein levels, IVM exhibited no substantial impact on PCV2 production. These results highlight the complexity of Cap shuttling between the cytoplasm and nucleus involving multiple NTR family members.

## Results

### Cap nuclear entry is an active transport process

**Figure 1:**
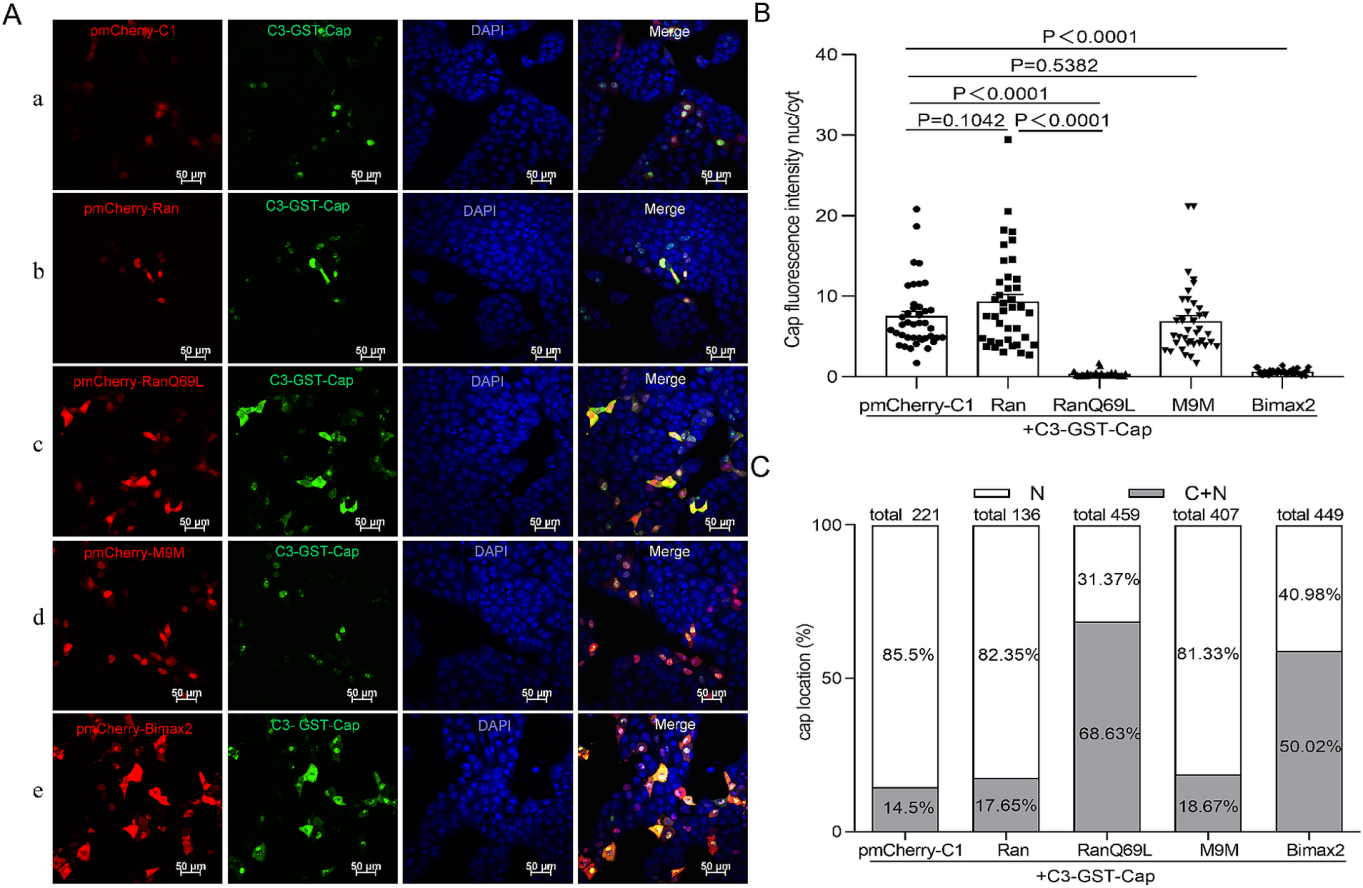
The nuclear import mechanism of C3-GST-Cap. **(A)** PK15 cells co-transfected with plasmids pEGFP-Cap-GST with pmCherry-C1, pmCherry-C1-Ran, pmCherry-C1-RanQ69L, pmCherry-C1-M9M, pmCherry-C1-Bimax2 for 24 h. Green fluorescence indicates cells expressing pEGFP-Cap-GST, while red fluorescence signifies cells expressing pmCherry fusion proteins. All photomicrographs were captured at a magnification of 400×, with a scale bar of 50 μM. **(B)** The Cap fluorescence ratio of nucleus to cytoplasm (N/C) was calculated for the selected regions of interest basing on each confocal microscopy image by ImageJ software, and 40 cells were chosen for statistical analysis under each condition, Data are presented as means ± SEM of three independent biological experiments. Statistical analysis was conducted using Student’s *t*-test. **(C)** The graph illustrates the percentages of cells with exclusive nuclear (N) distribution or with both cytoplasmic and nuclear (C + N) distribution of pEGFP-Cap-GST, based on the distribution of Cap in various treatment groups as shown in panel **(A)**.

**Figure 2:**
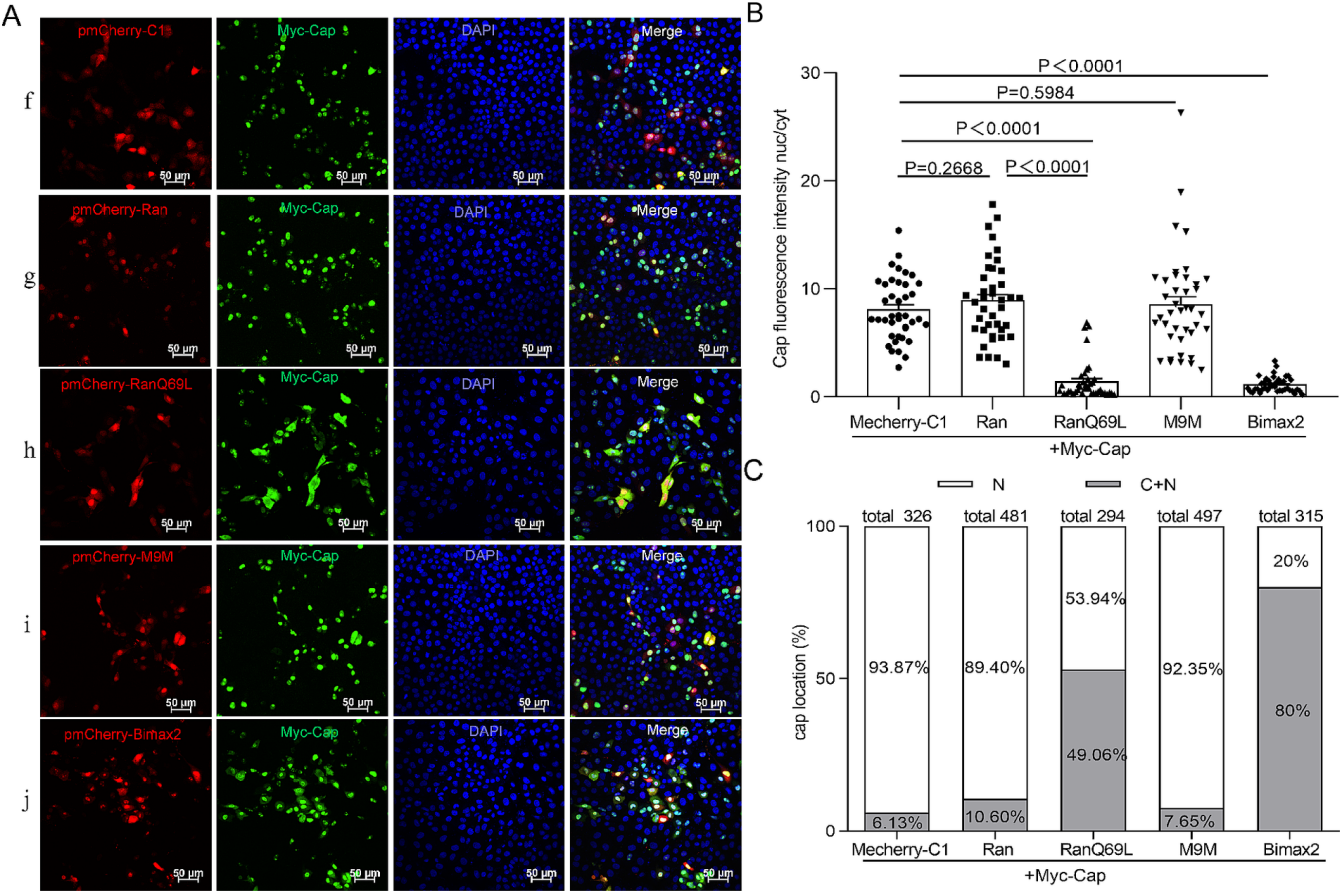
The nuclear import mechanism of Myc-Cap. **(A)** PK15 cells co-transfected with plasmids Myc-Cap with pmCherry-C1, pmCherry-C1-Ran, pmCherry-C1-RanQ69L, pmCherry-C1-M9M, pmCherry-C1-Bimax2 for 24 h. The resultant cells were fixed, incubated with mouse anti-Cap antibody, and then stained with anti-mouse FITC. Green fluorescence indicates cells expressing Myc-Cap, while red fluorescence signifies cells expressing pmCherry fusion proteins. All images were captured using the parameters specified in Figure 1. Scale bar, 50 μm. **(B)** The fluorescence ratio of nucleus to cytoplasm (N/C) was measured utilizing the same methodology detailed in Figure 1B, *N* = 40 cells for each condition. Data are presented as means ± SEM of three independent biological experiments. **(C)** The graph illustrates the percentages of cells with exclusive nuclear (N) distribution or with both cytoplasmic and nuclear (C + N) distribution of Myc-Cap, based on the distribution of Cap in various treatment groups as shown in panel **(A)**.

To date, while most studies have focused on the phenomenon of nuclear localization of Cap, none have systematically investigated its underlying transport mechanisms. PCV2 is a virus that replicates within the nucleus, and its mature virions are assembled from 60 identical capsid protein subunits. Previous research has indicated that viral particles and monomeric Cap proteins may utilize distinct pathways for nuclear import (Lin et al., 2022). To further investigate these differences, we constructed two eukaryotic expression plasmids encoding Cap proteins: C3-GST-Cap (molecular weight, MV > 50 kDa), which mimics the nuclear entry process of intact virions; conversely, myc-Cap (MW < 30 kDa), representing a monomeric form of the protein, was used to simulate the dynamics of nuclear import for smaller proteins. Ran has been implicated in NLS-dependent nuclear translocation as a GTPase enzyme that hydrolyzes GTP to GDP, providing energy for nuclear import (Li et al., 2015; Moore and Blobel, 1993). To explore the nuclear import mechanism of Cap, we introduced a dominant-negative mutant of RanGTP (Ran-Q69L), which is unable to hydrolyze GTP (Palacios et al., 1996), and co-transfected it with plasmids expressing either C3-GST-Cap or myc-Cap into PK15 cells for 24 h. Subsequently, the cells were fixed and incubated with specific antibodies to observe Cap localization. In RanQ69L-transfected cells, Cap fluorescence was observed in both the cytoplasm and the nucleus. In contrast, in the control groups co-transfected with pmCherry-C1 or wild-type Ran, Cap fluorescence was predominantly nuclear [Figure 1A (a, b, c) and Figure 2A (f, g, h)]. A significant reduction of the Cap fluorescence intensity nucleus-cytoplasm (nuc/cyt) ratio in Ran-Q69L-transfected cells, compared to pmCherry-C1 and pmCherry-Ran transfected cells, were observed (Figures 1B, 2B, *P* < 0.0001). Furthermore, We quantified the percentage of cells where Cap was localized exclusively to the nucleus (N) or partially in both the cytoplasm and nucleus (C + N). The results revealed that only 31.31% (C3-GST-Cap) and 37.76% (myc-Cap) of cells expressing Cap in RanQ69L co-transfected groups exhibited nuclear localization, compared to 82.35 and 89.4% in the wild-type Ran co-transfection groups with C3-GST-Cap or myc-Cap, respectively, and 85.5 and 93.87% in pmCherry-C1 co-transfection groups (Figures 1C, 2C). These findings demonstrate that Ran is required for the nuclear transport of Cap, suggesting that Cap employs an active transport process to enter the nucleus.

### Cap nuclear entry is an importin α-dependent process, but not mediated by transportin-1

To identify the cellular transporter responsible for Cap nuclear targeting, Bimax2 and M9M (designed as peptide inhibitors) were employed to effectively block classical import pathways: specifically, they target transport mediated by importin α and transportin-1, respectively (Cansizoglu et al., 2007; Kosugi et al., 2008). In the study, pmCherry-C1-Bimax-2 and pmCherry-C1-M9M were co-transfected with a Cap-expressing plasmid into PK15 cells, and the localization of Cap was observed under a fluorescence microscope. While M9M did not significantly inhibit Cap nuclear entry (~80–92% cells exhibit nuclear distribution), Bimax2 significantly and efficiently blocked the entry of Cap into the nucleus [Figure 1A (d, e), Figure 1B, Figure 2A (i, j), and Figure 2B, *P* < 0.0001], resulting in a substantial reduction of nuclear accumulation: only ~40–60 and 20% of cells, respectively, retained detectable nuclear signal for C3-GST-Cap and Myc-Cap following treatment (Figures 1C, 2C). Notably, immunofluorescence analysis revealed that control cells transfected with pmCherry-C1 vector (>85% cells) showed diffused nucleolar Cap staining (Figures 1C, 2C). These findings conclusively show that Cap nuclear entry occurs partially through the classical Importin α-dependent pathway but is independent of transportin-1-mediated transport.

### Cap binds with all importin-α isoforms and selected importin βs

It is widely recognized that cells across nearly all species utilize members of the importin family for subcellular transport. In vertebrates, three clades of α-type importins exist, encoding seven distinct human importin-α isoforms (importin α1/2, importin α3, importin α4, importin α5, importin α6, importin α7, and importin α8) (Sajidah et al., 2021). These function as adaptors connecting classical nuclear localization signals (cNLSs) with importin β (Sajidah et al., 2021). Importantly, the human genome encodes 20 Importin β family NTRs, among which ten are functional nuclear import receptors (NIRs): including importin β1/KapB1, transportin-1/IMPβ2, transportin-2/IMPβ2b, transportin-3/Trn SR, importin-4/RanBP4, importin-5/importin-β3/RanBP5, importin-7/RanBP7, importin-8/RanBP8, Importin-9/RanBP9, and importin-11/RanBP11 (Kimura and Imamoto, 2014; Kimura et al., 2017). These NTRs form multiple parallel transport pathways.

**Figure 3:**
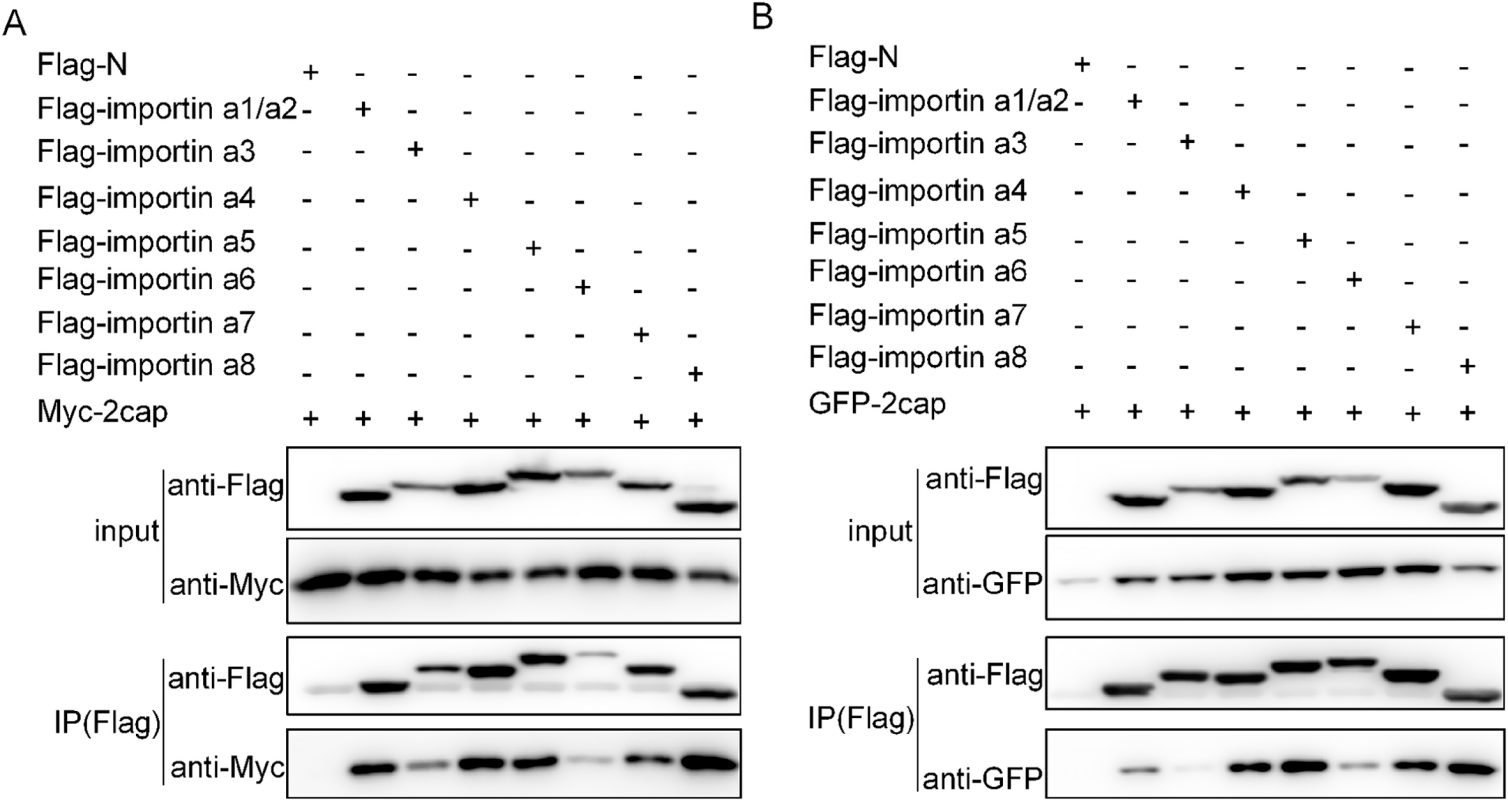
Verification of the interactions between Cap and importin α subunits. **(A)** Plasmids encoding Myc-Cap were co-transfected with the plasmids expressing seven Flag-tagged importin α subunits in 293 T cells for 36 h. **(B)** plasmids encoding pEGFP-C3-Cap were co-transfected with the plasmids expressing seven importin α subunits in 293 T cells for 36 h. Cell lysates were immunoprecipitated using Flag-agarose beads and the Cap protein was detected using anti-Myc pAbs or anti-GFP pAbs.

**Figure 4:**
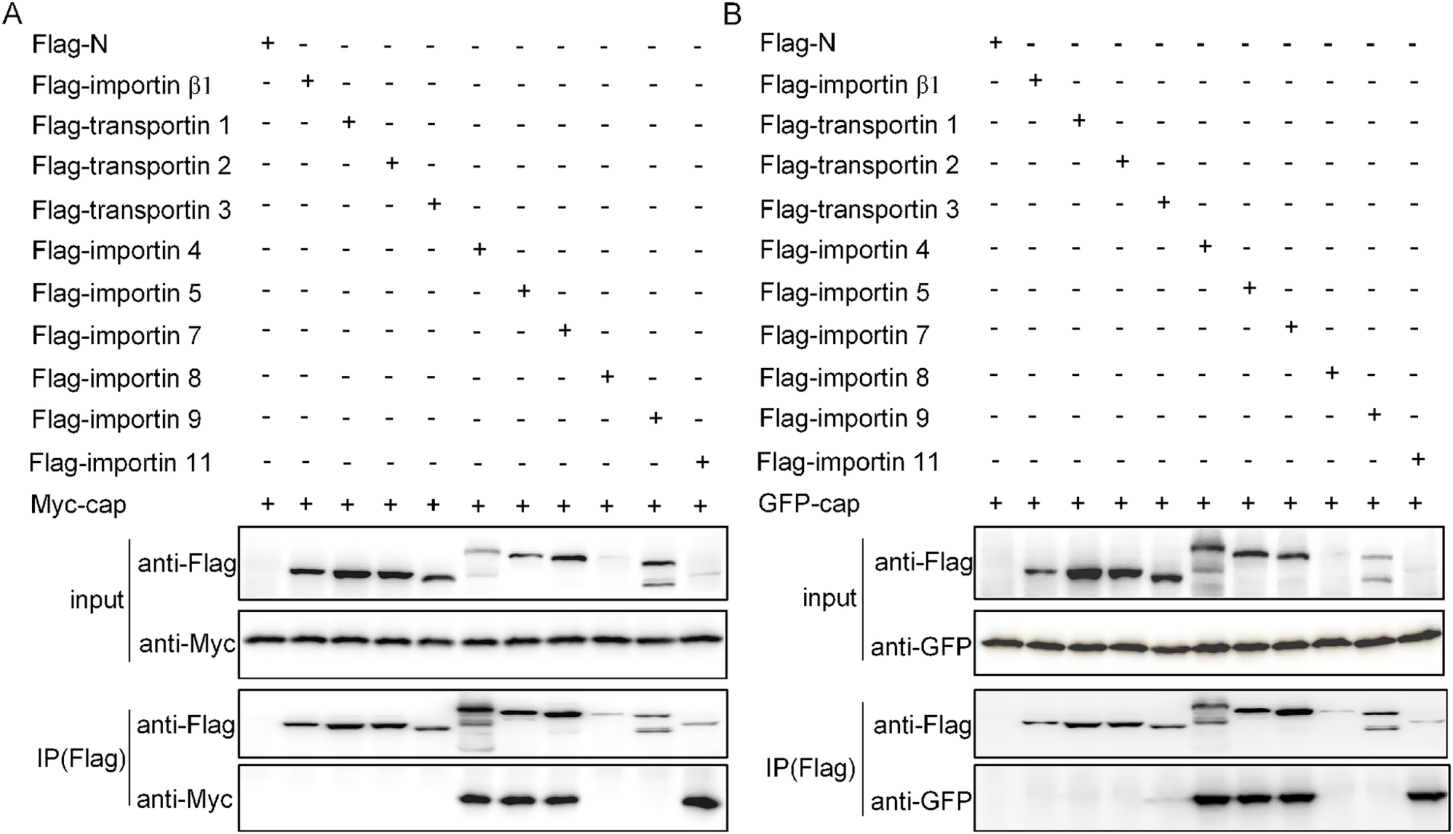
Verification of the interactions between Cap and transport subunits. **(A)** Ten nuclear import receptors were co-transfected with the Myc-Cap expression plasmid into 293 T cells for 36 h. **(B)** Additionally, plasmids encoding pEGFP-C3-Cap were co-transfected with plasmids expressing 10 nuclear import receptors in 293 T cells for the same duration. Cell lysates were then collected, immunoprecipitated using Flag-agarose beads, and subsequently immunoblotted with anti-Flag mAb, anti-Myc polyclonal antibody (pAb), and anti-GFP pAb.

To further validate our hypothesis regarding Cap nuclear import mechanisms, we systematically investigated its interactions with various importins. Co-transfection of HEK293T cells with flag-tagged plasmids expressing either Myc-Cap or C3-Cap, along with constructs encoding all seven importin-α isoforms and ten key NIRs (Importin β1/Karyopherinβ1, transportin-1/Importin β2, etc.), enabled us to identify specific binding partners. Using anti-Flag immunoprecipitation followed by immunoblotting with Myc, Flag, and GFP antibodies at 36 h post-transfection, we found that Cap specifically binds to all Importin-α isoforms, exhibiting a higher affinity for importin α1/α2, α4, α5, α7, and α8 (Figures 3A,B). Additionally, Cap also binds with four importin β family NTRs, which are importin 4, importin 5, importin 7, and importin 11 (Figures 4A,B). This indicates that Cap might utilize multiple nuclear import pathways facilitated by these binding importin subunits.

### Importazole inhibits PCV2 replication by blocking the importin β-mediated nuclear import of Cap

**Figure 5:**
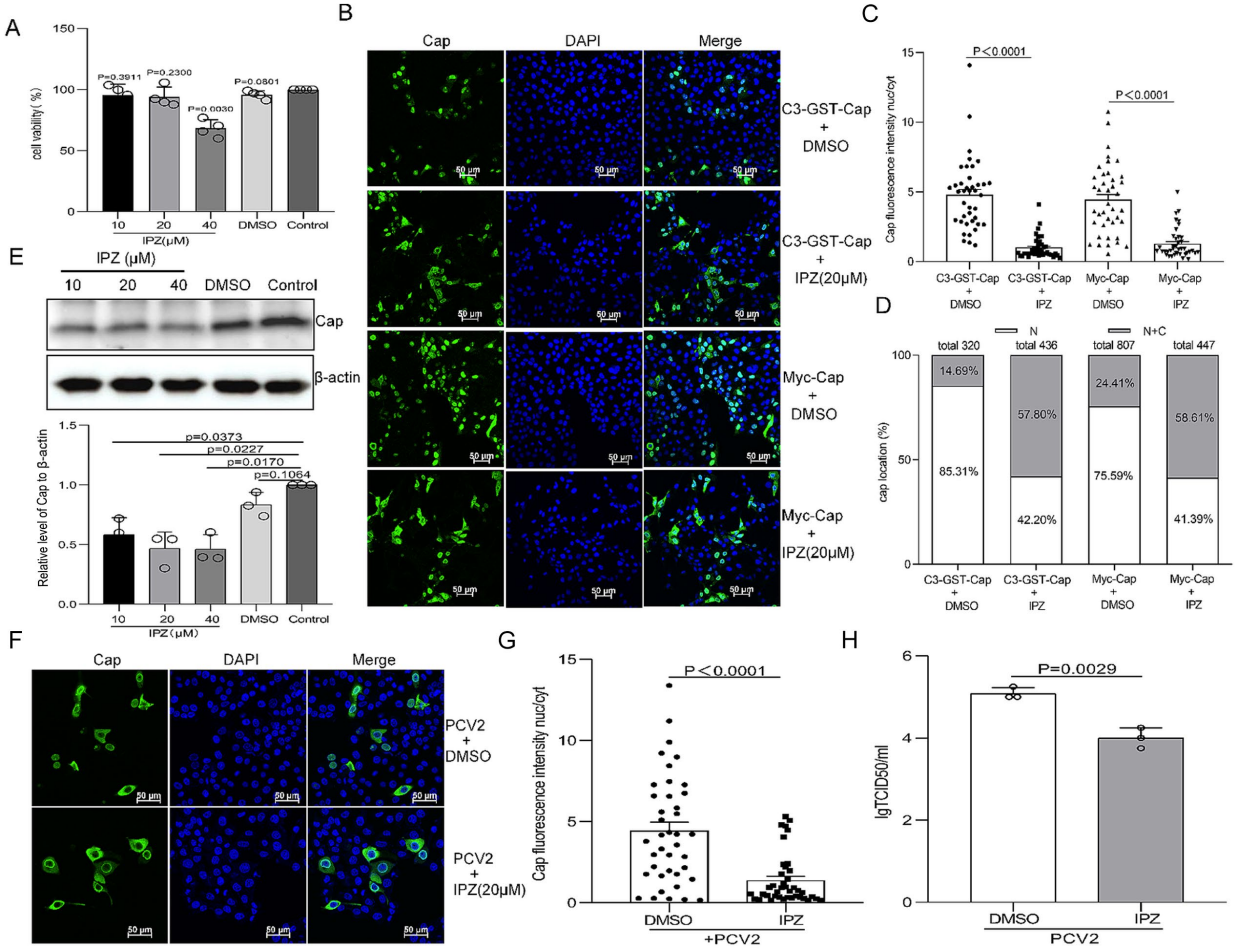
Importazole (IPZ) blocks Importin-β-dependent nuclear localization of Cap and inhibits the production of infectious PCV2 progeny. **(A)** The viability of PK15 cells pretreated with 10, 20, or 40 μM of importazole was analyzed using a CCK-8 assay. **(B)** Confocal dish-cultured PK15 cells were transfected with pEGFP-GST-Cap or Myc-Cap. At 8 h post-transfection, cells were treated with dimethyl sulfoxide (DMSO) or IPZ (20 μM). The cells were fixed at 24 h post-transfection. The distribution of Cap was detected with an anti-Cap antibody and observed by confocal microscopy. Scale bar, 50 μm. **(C)** The methodology used in this panel was the same as Figures 1B, 2B. **(D)** The graph depicts the percentages of cells exhibiting exclusive nuclear (N) localization or a combination of cytoplasmic and nuclear (C + N) localization of pEGFP-Cap-GST or Myc-Cap, based on the distribution of Cap within various treatment groups as depicted in panel **(B)**. PK15 cells were infected with PCV2 at an MOI of 1 and treated with the indicated concentration of IPZ, cells were harvested at 24 hpi. **(E)** Viral protein synthesis was assayed by western blotting using antibodies against the Cap protein, with β-actin serving as a loading control. WB bands were quantified using densitometry by ImageJ software and normalized against β-actin. The data are presented as means ± standard deviations (SD). Statistical analysis was conducted using Student’s *t*-test. **(F)** The resultant cells were fixed, incubated with mouse anti-Cap antibody, and then stained with anti-mouse FITC. All images were captured using the parameters specified in Figure 1A. Scale bar, 50 μm. **(G)** The fluorescence ratio of nucleus to cytoplasm (N/C) was measured as Figure 1B. **(H)** PK15 cells were infected with PCV2 at a multiplicity of infection (MOI) of 1 and treated with the indicated concentration of IPZ for 12 h. Cells were harvested at 24 hpi, and viral titers were determined. The results are presented as the mean TCID50 ± standard deviation (SD) (*n* = 3). Three independent biological experiments were performed.

To investigate the role of importin β-mediated nuclear transport in PCV2 replication, we employed IPZ, a specific inhibitor targeting importin β-RanGTP interactions (Soderholm et al., 2011). Cell viability assessments via CCK-8 assay revealed that IPZ pretreatment at 40 μM significantly reduced cell viability (Figure 5A, *P* < 0.05). However, concentrations of 10 and 20 μM did not show significant differences from mock-treated cells (Figure 5A, *P* > 0.05). Consequently, 20 μM IPZ was chosen for subsequent experiments. To assess the effect of IPZ on nuclear import of Cap, PK15 cells were treated with dimethyl sulfoxide (DMSO) or 20 μM IPZ post-transfection for 8 h. Confocal microscopy coupled with nuclear-to-cytoplasmic fluorescence intensity ratio analysis demonstrated that IPZ treatment significantly increased cytoplasmic fluorescence signals of Cap compared to DMSO controls (Figures 5B,C, *P* < 0.0001). Moreover, Quantification revealed significantly increased cytoplasmic distribution (>50% cell population) in IPZ-treated cells, and reduced cytoplasmic localization (8.75–21.86%) under control conditions (Figure 5D).

Furthermore, PK15 cells were infected with PCV2 at an MOI of 1 for 12 h, followed by IPZ treatment (20 μM) for an additional 12 h. Immunoblot analysis showed decreased Cap protein levels in IPZ-treated samples compared to DMSO controls and mock-infected groups (Figure 5E, *P* < 0.05). To further analysis if the reduction in Cap expression following IPZ administration was associated with decreased of PCV2 Cap nuclear transport. The localization of Cap was analyzed in 24-h PCV2-infected cells, which were either treated or not treated with IPZ at a concentration of 20 μM, using a Cap antibody by confocal microscopy. Consistent with the results of Cap-transfected cells, the cytoplasmic fluorescence signals of Cap were significantly increased in IPZ-treated cells compared to those treated with DMSO (Figures 5F,G, *P* < 0.0001). Moreover, the replication ability of PCV2 in IPZ-treated cells was almost 10-fold lower than that in the control cells (Figure 5H, *P* < 0.05). These findings collectively demonstrate that importin β-mediated nuclear import of Cap is required for PCV2 production.

### Ivermectin inhibits the nuclear import of Cap but does not affect infectious PCV2 progeny production

**Figure 6:**
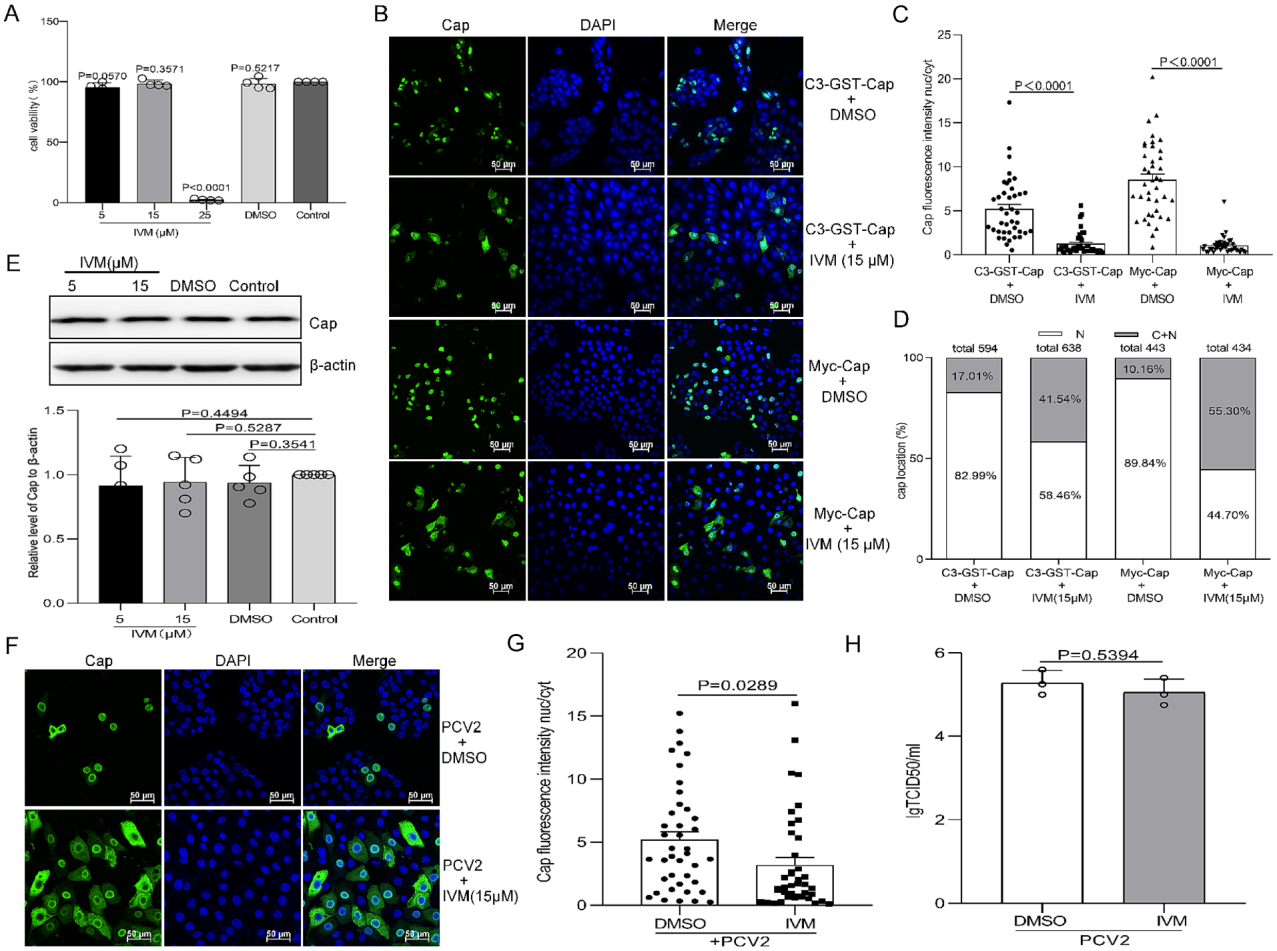
Ivermectin (IVM) inhibits Imp-α-mediated nuclear localization of Cap but does not affect the production of infectious PCV2 progeny. **(A)** The viability of PK15 cells pretreated with 10, 15, or 25 μM of IVM was analyzed using a CCK-8 assay. **(B)** Confocal dish-cultured PK15 cells were transfected with pEGFP-Cap-GST or Myc-Cap. At 8 h post-transfection, cells were treated with dimethyl sulfoxide (DMSO) or IVM (15 μM). The cells were fixed at 24 h post-transfection. The distribution of Cap was detected with an anti-Cap antibody and observed by confocal microscopy. Scale bar, 50 μm. **(C)** The methodology used in this panel was the same as Figures 1B, 2B. **(D)** The graph shows the percentages of cells displaying exclusive nuclear (N) localization or a combination of cytoplasmic and nuclear (C + N) localization for pEGFP-Cap-GST or Myc-Cap. The classification is based on the distribution of Cap across various treatment groups, as depicted in Panel B. PK15 cells were infected with PCV2 at an MOI of 1 and treated with the indicated concentration of IVM, cells were harvested at 24 hpi. **(E)** Viral protein synthesis was assayed by western blotting (WB) using antibodies against the Cap protein, with β-actin serving as a loading control. The WB bands were quantified as shown in Figure 5E. The data are presented as means ± standard deviations (SD). Statistical analysis was conducted using Student’s *t*-test. **(F)** The resultant cells were fixed and treated as shown in Figure 5F. **(G)** The fluorescence ratio of nucleus to cytoplasm (N/C) was measured as Figure 1B. **(H)** PK15 cells were infected with PCV2 and treated with IVM at a concentration of 15 μM for 12 h. Subsequently, the cells were harvested, and viral titers were detected, as indicated in Figure 5H.

IVM binds to the importin α armadillo (ARM) repeat domain, affecting thermal stability and α-helicity, preventing binding to importin β (Yang et al., 2020). To determine whether IVM influences Cap nuclear import and has antiviral effects against PCV2. First, cell viability was assessed using the CCK8 assay at various concentrations of IVM treatment (0–25 μM). The results showed no significant difference in cell viability between cells treated with 5 μM or 15 μM IVM compared to DMSO-treated controls for the same durations (Figure 6A, P>0.05), indicating low cytotoxicity at these concentrations and leading to the selection of 15 μM as the optimal working concentration. To assess the effect of IVM on Cap nuclear import, PK15 cells were treated with DMSO or 15 μM IVM post-transfection for 8 h. Confocal microscopy and Cap fluorescence intensity nucleus-cytoplasm (nuc/cyt) analysis revealed that treatment with IVM resulted in a significant increase in cytoplasmic fluorescence signals compared to DMSO-treated controls (Figures 6B,C, *P* < 0.0001). Quantification showed that above 40% of cells treated with IVM exhibited partial retention of green fluorescence, whereas only approximately 10–20% of control cells displayed this distribution pattern upon statistical analysis (Figure 6D). These data demonstrated that IVM treatment delays Cap nuclear import.

Furthermore, PK15 cells were infected with PCV2 at an MOI of 1 for 12 h followed by treatment with 15 μM IVM or DMSO for additional 12 h. Interestingly, the IVM enhances the cytoplasmic accumulation of Cap in PCV2-infected cells (Figure 6F), resulting in higher cytoplasmic fluorescence intensity than DMSO treatments. Cap fluorescence intensity nucleus-cytoplasm (nuc/cyt) analysis showed a significant difference between PCV2-infected cells treated with DMSO and those treated with IVM (Figure 6G, *P* < 0.0001). However, immunoblot analysis showed no significant difference in Cap expression levels despite the inhibition of Cap nuclear import (Figure 6E, *P* > 0.05). The replication ability of PCV2 in IVM-treated cells does not significantly differ from that of control cells (Figure 6H, *P* > 0.05). This indicates that IVM treatment causes the nuclear entry of the Cap protein, exerting no effect on viral titer.

## Discussion

PCV2 is a primary pathogen causing PMWS in pigs (Hamel et al., 1998). Mature PCV2 virions are formed by icosahedral Capsids composed of 60 identical Cap protein subunits arranged in a T = 1 surface lattice, with each of the viral particles’ outer shells consisting solely of capsid (Crowther et al., 2003; Tischer et al., 1982). The Cap protein plays critical roles in host immunity evasion, viral pathogenicity, and possesses indispensable role in viral life cycle (Blanchard et al., 2003; Fenaux et al., 2004; Fenaux et al., 2003; Wang et al., 2007; Wang et al., 2006). During both infection and transfection events, Cap protein undergoes rapid nuclear targeting, exhibiting distinct nucleocytoplasmic shuttling dynamics. It localizes diffusely in the nucleoplasm or punctate within the nucleolus before returning to the cytoplasm later (Cheung and Bolin, 2002; Finsterbusch et al., 2005; Lin et al., 2022; Liu et al., 2001). The subcellular localization patterns of Cap may thus be linked to various cellular processes (Finsterbusch et al., 2005). Despite decades of research on PCV, the precise mechanisms governing virion nuclear entry and monomeric Cap protein import remain incompletely understood. PCV2 particles have a diameter of just 17 nanometers. Recent research has tentatively confirmed that this virus enters the nucleus as intact viral particles (Lin et al., 2022; Wang et al., 2019). Similar mechanisms related to the nuclear entry of intact viral particles are observed in both the hepatitis B virus (HBV) and specific baculovirus capsids (Cohen et al., 2011). The HBV capsid protein relies on Importin α/Importin β-mediated transport to facilitate viral nuclear entry (Kann et al., 1999). In the present study, two eukaryotic expression plasmids, namely C3-GST-Cap and Myc-Cap, were constructed to simulate the nuclear entry processes of viral particles and the monomeric Cap protein. The results indicated that the nuclear entry efficiency of both plasmids was affected by the expression of RanQ69L and Bimax2, yet not by the expression of M9M. This implies that both viral particles and monomeric Cap protein are subjected to active transport mediated by importin α. Upon comparing the nuclear import data of two plasmids, it was observed that, in contrast to the monomeric Myc-Cap protein, the nuclear import efficiency of the C3-GST-Cap protein decreased significantly under the conditions of Bimax2 and RanQ69L overexpression. This phenomenon might imply that a higher degree of protein conformational complexity elevates the sensitivity to inhibitors. Upon competitive inhibition of importin α function by Bimax2, the interference with the nuclear import of C3-GST-Cap became more pronounced. This finding implies that the interaction interfaces between importin α and the two different forms of the Cap might vary, and the specific spatial structure formed by the polymer is more favorable for binding to importin α. Exploring these disparate modes not only offers novel experimental evidence for comprehending the nuclear entry mechanisms of PCV2 viral particles and Cap proteins but also enhances our understanding of the viral replication cycle and elucidates the pathogenicity mechanisms of PCV, including those associated with emerging strains like PCV3 and PCV4.

The nucleocytoplasmic transport system critically contributes to regulating cell division, metabolism, gene regulation, and innate immune responses (Dewi et al., 2021; Gu et al., 2016; Nakano et al., 2010; Perera et al., 2015; Sun et al., 2019; Tran and Wente, 2006). Most substances enter the nucleus via active or passive transport mechanisms, with RanGTP, NTRs, and NLS serving as critical components for active nuclear protein import (Sorokin et al., 2007). Specifically, Ran-GTP hydrolysis provides energy essential for GTP-dependent nuclear protein import (Palacios et al., 1996). In this study, the expression of the dominant-negative mutant RanQ69L significantly reduced the nuclear import percentages for both C3-GST-Cap and myc-Cap proteins (Figures 1, 2), confirming that viral capsids and Cap monomers undergo an active, RanGTP-dependent transport process to enter the nucleus. Most nucleocytoplasmic transport is facilitated by multi-subunit NTRs (Kimura and Imamoto, 2014; Kimura et al., 2017; Sajidah et al., 2021). In particular, seven importin α subunits and ten importin β homologs are involved in effective nuclear import. The Importin-α/β pathway serves as the part of nuclear transport mechanism, facilitating the entry of numerous viral proteins into the nucleus. Dengue virus (DENV) NS3 has been shown to possess an NLS that facilitates the nuclear transport of the protein via canonical Importin-α/β pathway (Palacios-Rapalo et al., 2021; Palacios-Rapalo et al., 2023). Recent investigations demonstrate that Zika virus (ZIKV) NS3 utilizes an identical mechanism for nuclear import, as evidenced by IVM-mediated transport inhibition studies which implicate involvement of the classical Importin-α/β machinery in facilitating ZIKV NS3 nuclear entry (De Jesus-Gonzalez et al., 2023). The PCV2 Cap is no exception, it binds simultaneously to multiple Importin α and -β homologs (Figures 3, 4). Furthermore, the nuclear import of the Cap protein is inhibited by IVM (Figures 6B,F), indicating the utilization of the Importin-α/β pathway for Cap nuclear entry. Besides, the canonical pathway, Multiple viral proteins interact with receptors via diverse NLS to leverage various entry routes (Sajidah et al., 2021). For instance, HPV16 L2 protein efficiently translocates to the nucleus of infected cells to facilitate assembly of HPV virions by binding to the Kapβ2, Kapβ3, and Kapα/Kapβ1 complexes (Darshan et al., 2004). In this study, numerous important β homologs were shown to bind with Cap. Based on the current comprehension of nuclear transport mechanisms, it is hypothesized that Cap might be transported through the NLS sequence (amino acids 1–41), which is recognized by the classical Importin-α/β heterodimer, rather than via the PY-NLS/transportin 1 pathway. Apart from the classical import route, Cap may also employ non-classical nuclear entry pathways, possibly mediated by Importin-β independently. The relative precedence of these mechanisms remains to be determined. From a viral standpoint, employing diverse import strategies likely enhances evasion from host cellular controls during nuclear transport, supports efficient replication, and contributes to viral adaptation and survival.

NTRs are frequently exploited by viruses to hijack host cellular machinery. For instance, influenza A virus (IAV) utilizes specific importin pathways—such as importin-α/β1—for nuclear localization of its NP protein and assembly of viral ribonucleoproteins (vRNPs), thereby promoting replication (Gabriel et al., 2008; Luo et al., 2018). Additionally, several viruses evade immune responses by competitively inhibiting transcription factors from accessing NTRs, examples include Severe Acute Respiratory Syndrome Coronavirus-2 (SARS-CoV-2) ORF6 and Ebolavirus (EBV) VP4 proteins blocking interferon-stimulated genes like Signal Transducer And Activator Of Transcription 1 (STAT1) or Interferon regulatory Factor 3 (IRF3) to counteract antiviral effects (He et al., 2017; Miorin et al., 2020; Reid et al., 2006). In this study, we identify multiple host importins interacting with PCV2 Cap protein. Notably, importin 5 binds specifically to the nuclear localization signal within Cap, shielding it from host degradation and facilitating the nuclear import of newly invading PCV2 capsids (Lin et al., 2022). Intriguingly, preliminary data from our laboratory concerning the role of importin 4 in PCV2 infection suggest that importin 4 promotes viral propagation without enhancing the nuclear translocation of the Cap. These findings highlight distinct roles among these importins in mediating Cap functions during infection. While multiple importin homologs interact with Cap, their specific contributions to nuclear transport versus replication support remain unclear. Further investigation is warranted to precisely determine which NTR-mediated mechanisms are essential for productive PCV2 infection and how they might be targeted therapeutically.

Current therapeutic strategies targeting viral nuclear transport proteins primarily involve the synthesis of peptide or peptidomimetic compounds designed to mimic virus-host transporter binding interactions. These approaches aim to reduce viral load and/or alleviate clinical symptoms, thereby achieving antiviral effects (Sajidah et al., 2021). Importin-α/β heterodimers represent key universal NTRs responsible for nuclear import across diverse viruses. Specific inhibitors targeting Importin-α have demonstrated efficacy against various pathogens. For instance, Bimax 2 (Kosugi et al., 2008), ivermectin (Tay et al., 2013; Wagstaff et al., 2012), cSN50.1 (Zienkiewicz et al., 2013), and GW5074 (Yang et al., 2019) all function by preventing cargo loading or disrupting the formation of importin-α/β heterodimers. Notably, IVM exhibits broad-spectrum antiviral activity against both RNA and DNA viruses *in vitro* and *in vivo* (King et al., 2020; Low et al., 2022; Yang et al., 2020). Similarly, importazole and M9M are known importin-β inhibitors with documented efficacy in cancer cell models (Cansizoglu et al., 2007; Soderholm et al., 2011). In our study, we discovered that importin-α inhibitors (Bimax 2 and IVM) and the Importin-β inhibitor IPZ effectively block Cap nuclear import (Figures 1A–E, 2A–J, 5B,F, 6B,F). This discovery prompted us to consider the integration of IVM and IPZ into anti-PCV2 strategies. Nonetheless, experimental investigations have demonstrated that both drugs can effectively inhibit Cap nuclear entry, however, only IPZ significantly reduces viral replication (Figures 5H, 6H). We postulate that IVM does not influence viral replication due to a potential compensatory mechanism and/or immune suppression. As is widely recognized, Importin α functions as a linker protein, binding to specific cargo and importin β to form the “cargo - linker - transporter” complex (Goldfarb et al., 2004). Besides this model, the “cargo - transporter” transport mechanism also exists within the transportation system (Kimura and Imamoto, 2014). IVM specifically inhibits the direct binding between importin α and β (Yang et al., 2020). Consequently, subsequent to IVM treatment, cells may activate compensatory mechanisms via alternative transport pathways. This is corroborated by our research, which reveals that the Cap protein interacts with multiple transport proteins. Additionally, the literature indicates that Importin α promotes the nuclear entry of immune factors, such as IRF3, during PCV2 infection (Li J. et al., 2018; Zhang et al., 2020). The blockade of the importin α/β pathway by IVM may also affect the nuclear entry of immune-related factors, potentially attenuating cellular antiviral responses. In contrast, IPZ directly and effectively inhibits the core transport step (Importin β - RanGTP), thereby successfully preventing viral proteins from entering the nucleus and replicating, which demonstrates its antiviral efficacy. Despite the promising broad-spectrum activity observed for several compounds against PCV2 in our initial screens, their potential impact on essential cellular transport functions raises concerns regarding possible side effects. Therefore, future research efforts should focus on developing virus-specific nuclear transport inhibitors that maintain selective efficacy while preserving normal.

## Materials and methods

### Cells and viruses

The porcine kidney epithelial cell line (PK15) (CCL-33, ATCC, Manassas, VA, United States) and the human embryonic kidney epithelial cell line (HEK 293 T) (CRL-3216, ATCC) were cultured in Dulbecco’s modified Eagle’s medium (DMEM, Servicebio) supplemented with 10% fetal bovine serum. The PCV2 JXAAS2306 (GenBank: PQ851824) strain was isolated from porcine tissue disease material and preserved by our laboratory. After serial passage in PK15 cells, the 50% tissue culture infective doses reached 10^7^ [TCID50]/mL.

### Antibodies and reagents

Lipofectamine™ 2000 transfection reagent (11668019) was purchased from Thermo Fisher Scientific, ExFect transfection reagent (T101-01/02) was obtained from Vazyme Biotechnology (Nanjing, China). anti-Myc polyclonal antibodies (pAbs) (R1208-1), rabbit anti-GFP pAbs (ET1602-7) and Mouse anti-β-actin monoclonal antibodies (mAbs) (M1210-2) were purchased from Huaan Biological Technology (Hangzhou, China). Anti-FLAG affinity resin (A2220) and anti-Flag mAbs (F1804) was obtained from Sigma-Aldrich.

### Plasmid construction

The Cap gene, cloned from the PCV2 JXAAS2306 genome, was inserted into the specified plasmids, yielding pCMV-Myc-Cap, pEGFP-C3-Cap, and pEGFP-GST-Cap. The Bimax-2 (Kosugi et al., 2008) and M9M (Cansizoglu et al., 2007) were synthesized and inserted into the indicated plasmids, resulting in pmCherry-C1-Bimax-2 and pmCherry-C1-M9M by Shangya Biotechnology. The full-length Ran was amplified by PCR from the cDNA of PK15 cells and inserted into the plasmids pmCherry-C1 (Clontech), resulting in pmCherry-C1-Ran. The Ran mutant variant pmCherry-C1-RanQ69L was constructed using inverse PCR with pmCherry-C1-Ran as the template, PCR products purified, digested with DpnI, and transformed into *Escherichia coli* strain DH5α. All nuclear transport factors were amplified from the cDNA of PK15 cells and subcloned into pCMV-N-Flag (Clontech) to generate constructs such as pCMV-N-Flag-importin α1 to α8, pCMV-N-Flag-importin β1, pCMV-N-Flag-importin 4, 5, 7, 8, 9, 11 and pCMV-N-Flag-transportin 1, 2, 3. All constructs were confirmed by sequencing. All the primers used in this study are available upon request.

### Transfection and fluorescence microscopy

Transfection and fluorescence microscopy assays were conducted as previously described (Lin et al., 2018; Lin et al., 2022). In brief, PK15 cells were seeded into 35-mm-diameter glass bottom dishes and grown to 40 to 50% confluence. The specified plasmid DNA was mixed with Lipofectamine^™^ 2000 transfection reagent according to the manufacturer’s protocol and introduced into PK15 cells. Twenty-four hours post-transfection, cells were fixed with 4% paraformaldehyde for 20 min. Myc-Cap-expressing cells were then incubated with anti-Myc-pAbs as the primary antibody and fluorescein isothiocyanate (FITC)-conjugated goat anti-rabbit IgG (KPL) as the secondary antibody. Finally, nuclei were stained with 4′,6-diamidino-2-phenylindole (DAPI, Servicebio) for 10 min before examination under a Nikon Ti2-E confocal laser microscope (Nikon, Japan).

### Co-immunoprecipitation and immunoblotting

Co-immunoprecipitation (co-IP) and immunoblotting (IB) were conducted following previously established protocols (Lin et al., 2018; Lin et al., 2022). In brief, vectors encoding Flag-tagged nuclear transport factors were co-transfected with vectors encoding Myc-tagged Cap or GFP-tagged Cap, respectively. Cells were collected 36 h post-transfection and lysed on ice using NP-40 lysis buffer supplemented with 1 mM phenylmethylsulfonyl fluoride (PMSF) for 2 h. The supernatant was then incubated with Flag-agarose beads for a minimum of 4 h at 4 °C. The agarose beads were washed three times with NP-40 lysis buffer. Subsequently, the immunoprecipitated proteins and cell lysates were subjected to IB analysis using mouse anti-Flag mAbs, rabbit anti-Myc pAbs and rabbit anti-GFP pAbs. The protein bands were visualized with a Super Western ECL substrate (Biobest) and captured using a chemiluminescent imaging system AI800 (GE Healthcare).

### Cell viability assay

To prevent significant cytotoxicity from drug treatment that could directly influence experimental results and obscure the drug’s intrinsic effects, we treated cells with varying concentrations of the drug to evaluate cell viability. Following the guidelines of the Cell Counting Kit-8 (CCK8) assay kit, PK15 cells were passaged and seeded into 96-well plates at 100 μL per well (approximately 10^4 cells per well), followed by an overnight culture. Subsequently, the cells were treated with 10, 20, or 40 μM of IPZ or 5, 15, or 25 μM of IVM, while the control group received an equal volume of DMSO. The 96-well plates were incubated at 37 °C for 16 h. Then, 10 μL of CCK8 solution was added to each well, and incubation continued for another 2 h. During this time, the absorbance of each well was measured at 450 nm using a microplate reader. The readings at a specific time point were selected for analysis. Cell viability (%) was calculated using the following formula: (OD of the drug-treated group - OD of the blank group) / (OD of the control group - OD of the blank group) × 100%.

### Cell treatment

To detect the effect of IPZ (20 μM, Medchemexpress MCE) and IVM (15 μM, Medchemexpress MCE) on Cap localization, Cap plasmids were transfected into PK15 cells for 8 h, after which the cells were treated with or without IPZ or IVM for an additional 16 h. Subcellular localization of Cap was analyzed by indirect immunofluorescence assay (IFA) using an anti-Cap monoclonal antibody (mAb). To investigate the impact of IPZ or IVM on the virus’s replicative ability, varying concentrations of IPZ (10, 20, and 40 μM) or IVM (5, 15 μM) were added to PK15 cells 12 h post-infection (hpi) for a duration of 12 h. Cell lysates were then collected and subjected to immunoblotting using an anti-Cap mAb and an anti-β-actin mAb.

### Viral titers assay

To analyze the replicative ability of PCV2 in IPZ- or IVM-treated cells, 20 μM or 15 μM IVM were added to PK15 cells at 12 hpi for a duration of 12 h. The cells were harvested at the indicated times and freeze-thawed three times. Subsequently, the supernatant fraction was collected to perform the titration of the 50% tissue culture infective dose (TCID50). An immunofluorescence assay (IFA) was conducted according to a previously described protocol to determine the viral titer (Cao et al., 2015).

## Data Availability

The original contributions presented in the study are included in the article/supplementary material, further inquiries can be directed to the corresponding author.
